# Gut microbiota changes associated with low-carbohydrate diet intervention for obesity

**DOI:** 10.1515/biol-2022-0803

**Published:** 2024-01-27

**Authors:** Li Li, Xiaoguo Zhao, Rashidin Abdugheni, Feng Yu, Yunyun Zhao, Ba-Fang Ma, Zhifang Yang, Rongrong Li, Yue Li, Yasen Maimaitiyiming, Mayila Maimaiti

**Affiliations:** Clinical Nutrition Department of the First Affiliated Hospital of Xinjiang Medical University, Urumqi 830011, Xinjiang, China; School of Public Health, Xinjiang Medical University, Urumqi 830011, Xinjiang, China; State Key Laboratory of Desert and Oasis Ecology, Xinjiang Institute of Ecology and Geography, Chinese Academy of Sciences, Urumqi, China; Department of Immunology, School of Basic Medical Sciences, Xinjiang Medical University, Urumqi 830011, Xinjiang, China; Department of Public Health, and Department of Hematology of First Affiliated Hospital, Zhejiang University School of Medicine, Hangzhou 310058, Zhejiang, China

**Keywords:** low-carbohydrate diet, gut microbiota, obesity, weight loss, 16s rRNA sequencing

## Abstract

Low-carbohydrate diets (LCDs) are frequently recommended for alleviating obesity, and the gut microbiota plays key roles in energy metabolism and weight loss. However, there is limited in-human research on how LCD changes gut microbiota. In this before–after study, 43 participants were assigned to the LCD intervention for 4 weeks. The main objective was to investigate the specific changes that occur in the participants’ microbiome in response to the LCD. Changes in gut microbiota were analyzed using 16s rRNA sequencing. Body composition was measured using InBody 770. Remarkably, 35 participants (79.07%) lost more than 5% of their body weight; levels of BMI, body fat, and total cholesterol were significantly decreased, indicating the effectiveness of the LCD intervention. The richness of microbiota significantly increased after the intervention. By taking the intersection of ANOVA and linear discriminant analysis effect size (LEfSe) analysis results, we identified three phyla, three classes, four orders, five families, and six genera that were differentially enriched between baseline and week-4 time points. Among the three phyla, relative abundances of *Firmicutes* and *Actinobacteriota* decreased significantly, while *Bacteroidetes* increased significantly. At the genus level, *Ruminococcus*, *Agathobacter*, *Streptococcus*, and *Bifidobacterium* showed a significant reduction in relative abundances, whereas *Parabacteroides* and *Bacteroides* increased steadily. Our results demonstrate that LCD can effectively alleviate obesity and modify certain taxa of gut microbiota, providing potential insights for personalized dietary interventions against obesity.

## Graphical abstract

**Figure:**
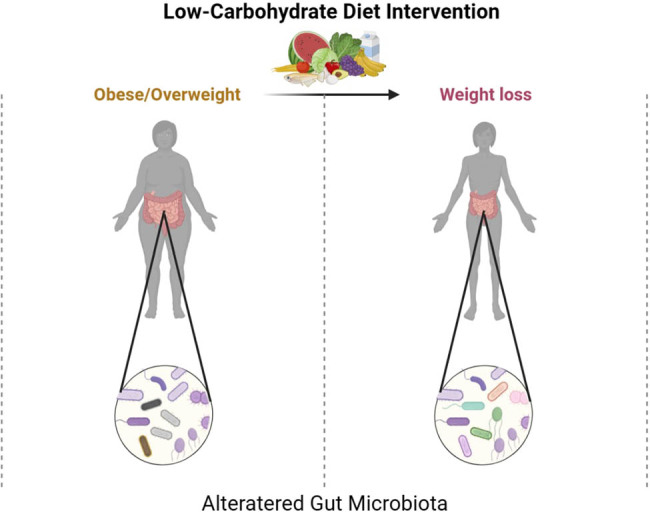

## 1 Introduction

Obesity, a global health problem, affects a significant number of people worldwide [1,2]. This complex disorder is characterized by an imbalance between energy intake and expenditure, leading to excessive body fat accumulation [3]. In recognition of World Obesity Day on March 4, 2022, the World Health Organization (WHO) reported staggering statistics: over 1 billion individuals, including 650 million adults, 340 million teenagers, and 39 million children, are classified as obese (BMI ≥ 30 kg/m^2^) globally [4]. It is more alarming to note that these numbers continue to increase. Obesity not only predisposes individuals to various health issues but also increases the risk of developing several disorders such as asthma, cardiovascular diseases, osteoarthritis, sleep disorders, type 2 diabetes mellitus, mental health issues, nonalcoholic fatty liver disease, and certain types of cancer, among others [5,6,7,8,9,10]. These obesity-related diseases are major contributors to premature or preventable deaths [11]. Consequently, extensive research has focused on investigating the factors contributing to obesity as well as developing effective intervention strategies to prevent its occurrence.

Obesity is believed to be influenced by a combination of factors, including genetic susceptibility, physiological and biological pathways related to metabolism and body weight regulation, physical activity and exercise choices, as well as dietary habits and environmental factors [1,2,3]. In response to this understanding, various strategies have been developed to address obesity. These include pharmacotherapy [12], surgical treatment [13], exercise therapy [9], and dietary interventions among others [14], all of which have pros and cons, respectively. Pharmacotherapy and surgical treatment are options for obesity management but come with inherent costs and potential risks [2,15]. In contrast, exercise therapy is a relatively economical option, but its effectiveness relies on long-term and consistent adherence, which can be challenging or even impossible for individuals who are overweight or obese [16]. In comparison, dietary interventions are currently considered a popular, conservative, and convenient strategy for treating and managing obesity. Such interventions are highly safe, cost-effective, and offer a promising approach when compared to other alternative strategies [14,17].

Among the various dietary patterns that have been studied for their effectiveness in weight loss and managing obesity-related comorbidities, the low-carbohydrate diet (LCD) stands out as a widely studied approach [18,19,20]. A study found that LCD intervention is superior to exercise in improving obesity [21]. Their results showed that after a 3-week intervention, the LCD group experienced greater weight loss (−3.56 ± 0.37 kg) compared to the exercise group (−1.24 ± 0.39 kg), as well as larger reductions in fat mass, waist circumference, and various glycemic parameters. While debates persist regarding the comparative effectiveness of LCDs versus low-fat diets (LFDs) or energy-restricted weight loss diets, there is a consensus that LCDs have a positive impact on reducing body mass index (BMI), body fat, waist circumference, blood pressure, serum lipid levels, insulin levels, etc. [22,23]. Inconsistencies observed in randomized controlled trials may be attributed to variations in dietary composition [24]. However, the individual variability in response to LCD within a trial highlights the need to consider other factors that influence weight loss.

Accumulating evidence suggests that the gut microbiota (GMB) plays a crucial role in obesity by influencing energy intake and nutrient absorption in the host [25]. The GMB contributes to obesity through various mechanisms. Dietary patterns have been found to influence the composition and diversity of the GMB [26]. Interestingly, the GMB also influences dietary intake through gut–microbiome–brain interactions, indicating a bidirectional relationship between diet and the GMB [27]. Animal studies have demonstrated that fecal transplantation from obese mice to germ-free mice results in weight gain and the development of obesity-related metabolic phenotypes, which can be alleviated by cohabitation with mice harboring the microbiota of lean co-twins [28]. When the germ-free mice were colonized with the conventional GMB, they notably gained weight and exhibited over 50% increased levels of body fat [29]. GMB can increase energy extraction by breaking down complex carbohydrates and producing fatty acids [30]. Gut bacteria also impact metabolism, influencing the absorption and storage of nutrients [31]. For instance, short-chain fatty acids (SCFAs), such as acetate, propionate, and butyrate, can promote fat storage, while trimethylamine and trimethylamine N-oxide from certain gut bacteria have been associated with increased obesity and metabolic dysfunction. Additionally, they regulate appetite and food cravings by affecting hormone production [32]. Imbalances in gut bacteria can promote inflammation and insulin resistance, further contributing to weight gain [33]. Taken together, these findings suggest that the GMB may serve as a mediator in the effects of LCD interventions on obesity [25]. However, there is limited research on the effects of LCDs on anthropometric profiles, body composition, and GMB in humans. Further exploration is needed to: (1) evaluate the impact of LCD on individuals’ health indicators, and (2) understand the influence of LCD on the composition and structure of the host’s gut microbiome. Thus, the main objective of this study was to investigate the specific changes that occur in the participants’ microbiome in response to the LCD.

In this study, we examined the effects of a 4-week LCD intervention on anthropometric profiles, body composition, and GMB of participants. The results demonstrated that the LCD intervention effectively reduced body weight (−6.14 kg [95% CI, −5.37 to −6.92 kg]; *p* < 0.001), BMI (−2.26 kg/m^2^ [95% CI, −1.96 to −2.56 kg/m^2^]; *p* < 0.01), body fat, (−4.73 kg [95% CI, −4.10 to −5.35 kg]; *p* < 0.001), visceral fat area (−21.25 cm^2^ [95% CI, −17.59 to −24.91 cm^2^]; *p* < 0.001), and serum total cholesterol levels. These findings indicate that the LCD intervention strategy was successful in treating obesity. Notably, we observed changes in the abundance of microbial phyla following the intervention. Specifically, there was an enrichment of *Bacteroidetes* (including genera *Parabacteroides* and *Bacteroides*), which have been associated with lower obesity risk. Conversely, there was a reduction in the abundance of *Firmicutes* (including genera *Ruminococcus*, *Agathobacter*, *Streptococcus*) and *Actinobacteriota* (now *Actinomycetota*) (including the genus *Bifidobacterium*), which have been linked to higher obesity risk. These findings suggest that our LCD intervention effectively modulated the composition of the participants’ GMB, providing a foundation for long-term digestive system health. In summary, this study demonstrated that LCD can effectively alleviate obesity and induce changes in specific gut microbial taxa. These observations may contribute to a better understanding of the mechanisms underlying LCD-mediated weight loss and offer potential insights for personalized dietary interventions against obesity.

## 2 Materials and methods

### 2.1 Study participants

The study was conducted in accordance with the Declaration of Helsinki and approved by the Ethics Committee of the First Affiliated Hospital of Xinjiang Medical University on June 22, 2020 (no. K202006-18). The study was implemented from July 2020 to June 2021. Overweight/obese but otherwise healthy participants, male and female, were voluntarily enrolled in the weight loss program (low-carbohydrate dietary guidance) of the Department of Clinical Nutrition, First Affiliated Hospital of Xinjiang Medical University. Written informed consents were taken from every participant prior to the study. All the participants have BMIs over 25 kg/m^2^ (33.43 kg/m^2^ on average). The exclusion criteria include consumption of alcohol or tobacco, participating in specific eating plans or excessive training programs at recruitment, undertaking any medication (prescribed drugs or antibiotics) treatment, and having an unstable weight (variation > 5%) in the past 6 months. As a result, a total of 61 participants were included in the study.


**Informed consent:** Informed consent has been obtained from all individuals included in this study.
**Ethical approval:** The research related to human use has been complied with all the relevant national regulations, institutional policies and in accordance with the tenets of the Helsinki Declaration, and has been approved by the Ethics Committee of the First Affiliated Hospital of Xinjiang Medical University on 22 June 2020 (no. K202006-18).

### 2.2 Study design, body composition, and metabolic parameters

This before–after study aimed to assess the changes in GMB among participants before and after a 4-week dietary intervention. All participants were enrolled in a single-arm study and assigned to a low-calorie diet intervention. Prior to the intervention, participants underwent 2 weeks of normal diet as a baseline. Subsequently, they switched to the LCD for 4 weeks. Trained dietitians maintained regular communication with participants through daily telephone calls or via a social app (WeChat) to ensure adherence to the LCD guidelines. During the study, fecal samples, body composition measurements, and blood tests were conducted at baseline and the end of the 4-week intervention period. Fecal samples were stored at −80°C prior to microbiome sequencing. Body composition measurements were performed using the InBody 770 at the outpatient clinic of the First Affiliated Hospital of Xinjiang Medical University. Out of the initial 61 participants, 43 returned for body composition measurements, blood tests, and fecal sampling. Therefore, all subsequent analyses related to body composition and GMB sequencing were conducted on these 43 participants. Detailed demographic data of the participants can be found in Table 1.

**Table 1:** Demographic characteristics of study participants and changes in anthropometric profiles after LCD intervention

Parameters	Baseline (*n* = 43)	Week 4 (*n* = 43)	*p* value
Female/male	28/15	28/15	
Age, years	31.51 (±10.50)	31.51 (±10.50)	
Weight	92.01 (±18.83)	85.87 (±17.88)	0.000
BMI	33.43 (±5.20）	31.17 (±4.83)	0.000
Ethnicity	36	36	
Han	4	4	
Uyghur	2	2	
Hui	1	1	
Kazakh	1	1	
Waistline (cm)	107.17 (±13.10)	99.20 (±13.07)	0.000
Hip circumference (cm)	113.88 (±9.63)	108.48 (±9.85)	0.000
Waist-to-hip fat ratio	0.99 (±1.52)	0.96 (±0.08)	0.005
Body fat (kg)	38.70 (±11.17)	33.97 (±10.75)	0.000
Skeletal muscle (kg)	29.67 (±6.92)	33.97 (±10.75)	0.237
Defatted body weight (kg)	53.32 (±11.45)	51.89 (±11.37)	0.203
Protein (kg)	10.49 (±2.28)	10.23 (±2.27)	0.246
Body moisture content (kg)	39.13 (±8.35)	38.05 (±8.31)	0.188
Intracellular fluid (kg)	24.29 (±5.31)	23.67 (±5.26)	0.232
Extracellular fluid (kg)	14.85 (±3.06)	14.38 (±3.06)	0.132
Inorganic salt (kg)	3.68 (±0.82)	3.61 (±0.80)	0.312
Body fat percentage (%)	41.80 (±6.73)	39.23 (±7.64)	0.004
Basal metabolic rate	1521.40 (±247.20)	1459.32 (±312.46)	0.052
Visceral fat area (cm^2^)	175.89 (±49.11)	154.64 (±52.13)	0.000
Total cholesterol (mg/dL)	1.98 (±1.04)	1.05 (±0.31)	0.000
Triglyceride (mg/dL)	5.03 (±1.07)	4.76 (±1.04)	0.153
HDL-C (mg/dL)	1.13 (±0.25)	1.08 (±0.27)	0.359
LDL-C (mg/dL)	3.40 (±0.82)	3.36 (±0.89)	0.791

### 2.3 Dietary intervention (LCD)

Participants were asked to take LCD (carbohydrate 25%, protein 45%, fat 30%), with drinking water of at least 1.8 L/day. Daily caloric intake (kcal) = ideal body weight (kg) × (20–25) × 75%. Ideal body weight (kg) = height (cm) −105. The dietary choices were mainly composed of low-glycemic and high-protein foods. Taboo foods included rice flour and other high-starch foods, fruits, cakes and other sweets, wines, and beverages. Vegetable oil was chosen as the main cooking oil. Participants adopted commercialized meal replacement bars and dietary fiber powders to ensure the LCD structure. Specific dietary recommendations: for breakfast, one egg, one bag of meal replacement milkshake, and one bag of dietary fiber powder; for lunch, a meal replacement bar, 200 g of raw meat (steamed or boiled fish and shrimp were priorities), and 150 g of raw non-starch vegetables; for dinner, a meal replacement bar, 150–200 g of raw meat (steamed or boiled fish and shrimp were priorities), and 150 g of raw non-starch vegetables. Each bag of meal replacement milkshake contained 7.3 g carbohydrate, 14.4 g protein, and 1.8 g fat, weighing 28 g and providing 107.5 kcal of energy. Each meal replacement bar contained 28.6 g carbohydrate, 31.2 g protein, and 14.3 g fat, weighing 56 g and providing 189.71 kcal of energy. Each bag of dietary fiber powder weighed 8 g, providing 12.42 kcal of energy. The body weight and urine ketone of participants were closely monitored. The intervention period lasted for 4 weeks (28 days).

### 2.4 16S rRNA gene-based microbiome sequencing

The 16S rRNA gene (rDNA) sequencing was conducted by Biomarker Biotechnology Co., Ltd (Beijing, China). Following extraction of the total DNA from samples, specific primers with Barcode (27 F, 5′-AGRGTTTG ATYNTGGCTCAG-3′; 1492 R, 5′-TASGGHTACCTTGTTASGACTT-3′) were used to amplify the full-length sequence (V1–V9) of the 16S rRNA gene [34]. Then, the products were purified, homogenized, and quantified to form a sequencing library. After library quality inspection, the eligible library is sequenced with the PacBio Platform. The optimized circular consensus sequences (CCS) were obtained by filtering with the threshold of minPasses ≥5 and minPredictedAccuracy ≥ 0.9 [35]. After pre-processing the sequencing data, bioinformatics analysis is performed.

### 2.5 Bioinformatics analysis

In this study, all the bioinformatics analyses were performed using BMK Cloud (Biomarker Technologies Co., Ltd; www.biocloud.net). Sequences with >97% similarity were clustered into the same operational taxonomic unit (out) by USEARCH (v10.0) [36]. OTUs with an abundance of <0.005% were filtered. Taxonomic annotation of the OTUs was carried out based on the Naive Bayes classifier in QIIME2 (Version 1.8.0) using the SILVA database (release132) with a confidence threshold of 70% [37]. The alpha (*α*) and beta (*β*) diversities were calculated and visualized using the QIIME pipeline and R software. PCoA and PERMANOVA/ANOSIM analyses were performed for the *β* diversity estimation. The significant taxonomic differences were assessed by one-way ANOVA and the linear discriminant analysis (LDA) effect size (LEfSe). The logarithmic LDA score threshold was 3.5 for discriminative features.

### 2.6 Statistical analysis

Statistical analysis for comparison of the body composition and metabolic parameters was conducted using SPSS software (SPSS Statistics, v. 23). The results were expressed as mean ± SD (or SE), and differences were considered statistically significant at a *p* value < 0.05 (*p < 0.05; **p < 0.01; ***p < 0.001). Differences in demographic as well as anthropometric profiles, body composition, body fat distribution, and clinical and biochemical measurements were evaluated using paired *t*-test. One-way ANOVA analysis was performed at multiple taxonomic levels (phylum, class, order, family, genus, and species) to identify differentially enriched taxa between the two groups. Additionally, Benjamin and Hochberg’s false discovery rate method was used to correct and adjust *p*-values [38]. LEfSe with the default α value of 0.05 was carried out using BMKCloud (www.biocloud.net) to screen taxa that serve most possibly as biomarkers between the two time points.

## 3 Results

### 3.1 Effects of LCD on the body weight, body composition, and anthropometric profiles

**Figure 1:**
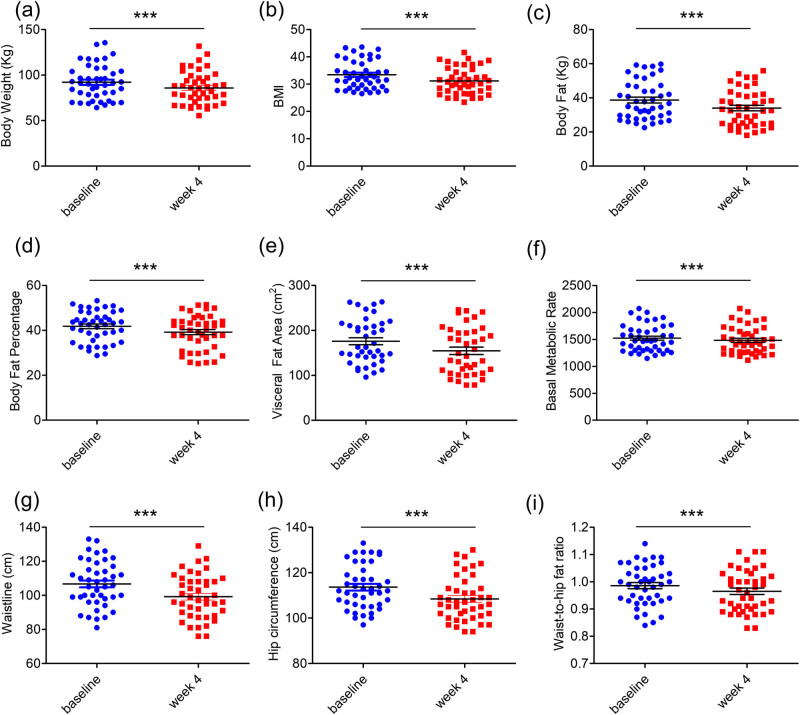
Scatter diagrams showing changes in body weight (a), BMI (b), body fat (c), body fat percentage (d), visceral fat area (e), basal metabolic rate (f), waisline (g), Hip circumference (h) and Waist-to-hip fat ratio (i) of participants after 4 weeks of LCD intervention.

After intervention with the LCD for 4 weeks, a significant reduction in the body weight (−6.14 kg [95% CI, −5.37 to −6.92 kg]; *p* < 0.001) and BMI (−2.26 kg/m^2^ [95% CI, −1.96 to −2.56 kg/m^2^]; *p* < 0.01) of the participants was noted (Figure 1a and b), which mainly resulted from a loss of body fat (−4.73 kg [95% CI, −4.10 to −5.35 kg]; *p* < 0.001) (Figure 1c and d). Notably, 34 of 43 (79.07%) participants gained a weight loss of at least 5% body weight, indicating the effectiveness of the intervention. The average visceral fat area of study participants was 175.89 cm^2^ at the baseline, and it decreased to 154.64 cm^2^ after 4 weeks of intervention (−21.25 cm^2^ [95% CI, −17.59 to −24.91 cm^2^]; *p* < 0.001) (Figure 1e). Other anthropometric profiles and body composition data including basal metabolic rate, waistline, hip circumference, and waist-to-hip fat ratio (Figure 1f–i) were also significantly decreased, along with a significant reduction in total cholesterol levels (Table 1). Collectively, the results of this study indicate that a 4-week LCD intervention leads to significant and meaningful anti-obesity effects. These findings provide strong evidence for the efficacy of LCD in promoting weight loss and combating obesity.

### 3.2 General profile of GMB composition

**Figure 2:**
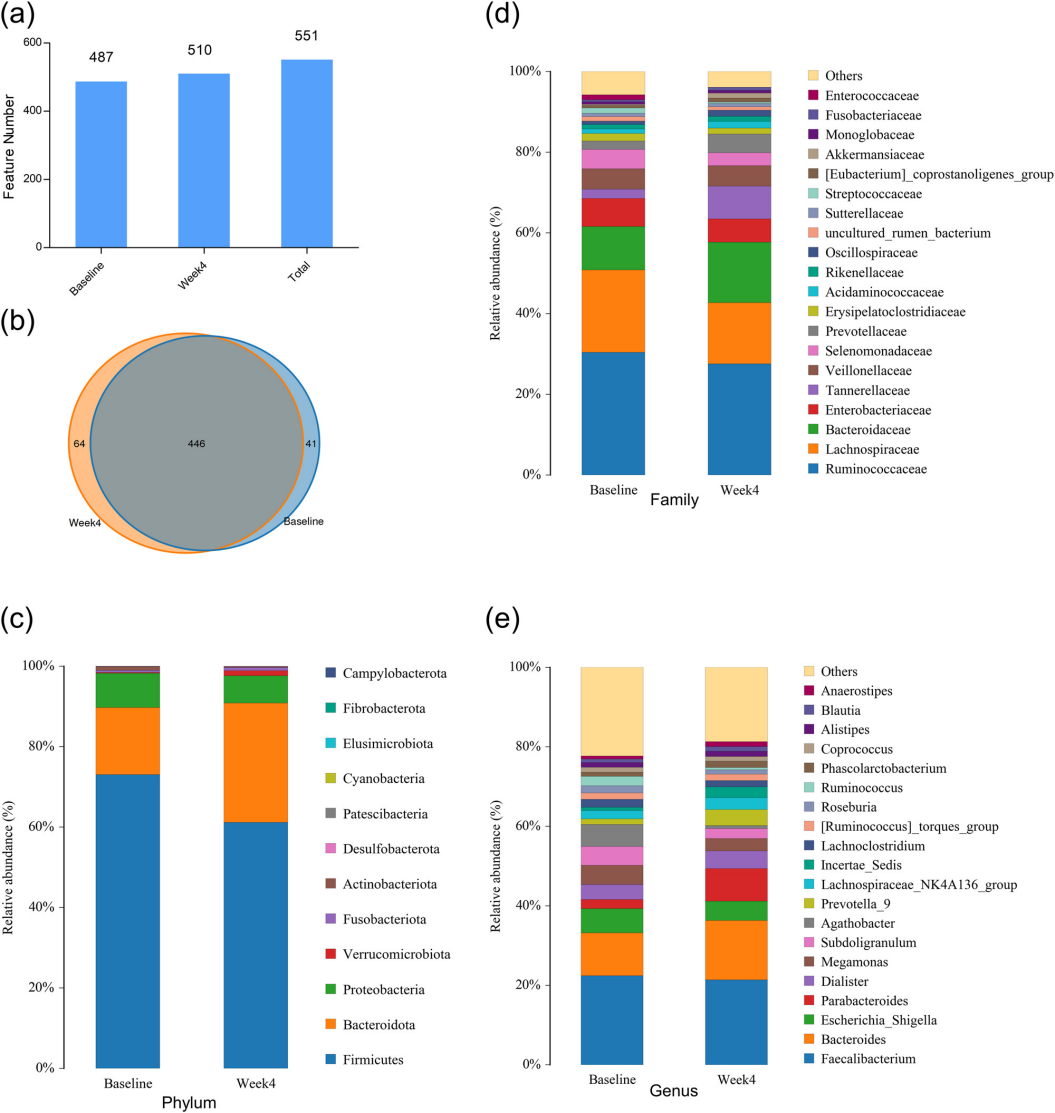
Overview of changes in multiple taxonomic levels by LCD intervention. (a) Bar chart showing OTU (feature) numbers at baseline and week 4. (b) Van diagram of OTUs at baseline and week 4. (c–e) Overview of microbiota levels at phylum, family, and genus levels at baseline and week 4.

A total of 86 fecal samples were sequenced and subjected to Barcode identification, which produced 580,075 CCS in total. Specifically, each sample produced at least 4,027 CCS, with an average of 6,745 CCS. The number of OTUs in each sample is shown in supplementary Figure S1. A total of 487 and 510 OTUs were found at the baseline and week 4, respectively, with a total of 551 OTUs in all samples (Figure 2a). Although most OTUs (464) overlapped between the two time points, there were 41 baseline-specific and 64 week-4-specific OTUs (Figure 2b). Taxonomic analysis revealed that these microbiotas belong to 12 phyla (Figure 2c). The relative abundance of the top 20 microbiome at family and genus levels are displayed in Figure 2d and e. Consistent with previous findings [27], our results showed that *Firmicutes* (now *Bacillota*) and *Bacteroidota* (previously known as *Bacteroidetes*) are the top two abundant bacterial phyla in the fecal samples and the *Faecalibacterium* is the most abundant genus (Figure 2c and e).

### 3.3 *α* and *β* diversity

**Figure 3:**
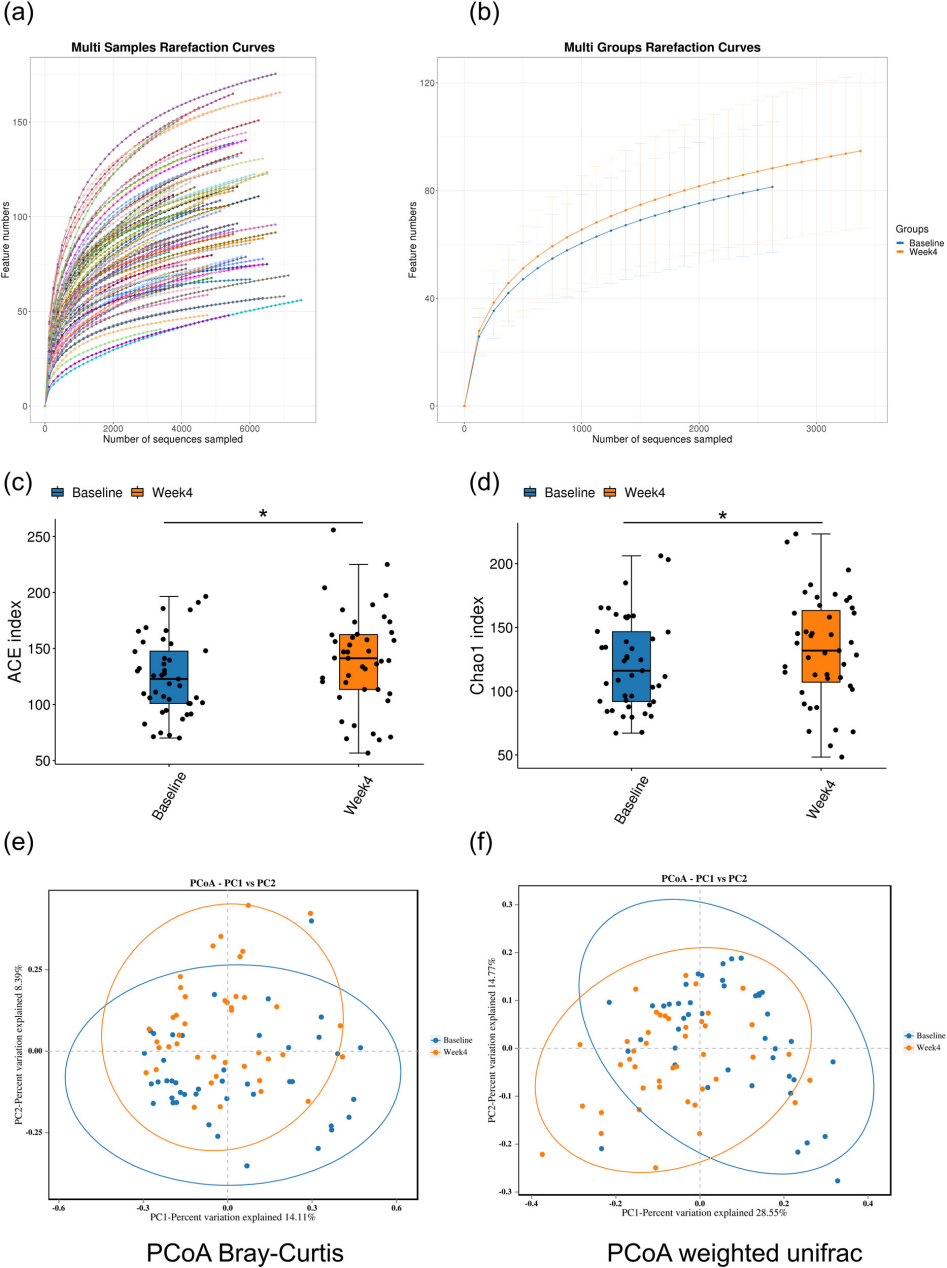
Changes in *α* and *β* diversity after the LCD intervention. (a and b) Rarefaction curves of all samples (86) as well as baseline and week 4 groups. (c and d) Box plots showing alterations in ACE and Chao1 indices. (e and f) Principal coordinates analysis (PCoA) of Bray–Curtis dissimilarity data and weighted unifrac.

*α* Diversity is a measure to evaluate the microbiota richness and diversity (or complexity) *within* samples. The rarefaction curve of each sample at baseline as well as week 4 were steeply increased and gradually reached a plateau, suggesting that the amount of sequencing data is sufficient to reflect the species diversity in each sample (Figure 3a and b). The abundance-based coverage estimator (ACE) and Chao 1 indices at week 4 were significantly increased as compared to that of the baseline, suggesting an increased richness of microbiota within each sample after 4 weeks of LCD intervention (Figure 3c and d). Intriguingly, while the Shanon and Simpson indices displayed a slight upward trend in the week-4 group, the differences compared to the baseline were not statistically significant (Figure S2a and b). This suggests that the diversity of the microbiota is not significantly increased following a 4-week intervention. *β* Diversity aims to evaluate the microbiota complexity between samples. Here, the PCoA analysis of Weighted Unifrac distance and Bray–Curtis dissimilarity revealed certain differences between the baseline and week-4 time points (Figure 3e and f). Further analysis of PERMANOVA/Anosim indicated a subtle inter-group but not intra-group differences (Supplementary Figure S2c–f), suggesting that the LCD intervention resulted in certain changes in GMB.

### 3.4 Alterations in the microbiota composition after LCD intervention

After a 4-week LCD intervention, significant changes in the composition of the GMB were observed through ANOVA analysis. In both baseline and week-4 time points, the top three phyla (*Firmicutes*, *Bacteroidota,* and *Proteobacteria*) constituted more than 97% of the total GMB (Table 2). Notably, among the top three phyla, the relative abundance of *Firmicutes* and *Bacteroidota* but not *Proteobacteria* (now *Pseudomonadota*) changed significantly after the intervention. Specifically, the relative abundance of *Firmicutes* was significantly reduced from 73.61% at baseline to 60.26% at week 4. In contrast, the relative abundance of *Bacteroidota* was significantly increased from 16.13% at baseline to 30.73% at week 4. Consistent with previous reports, the ratio of *Firmicutes* to *Bacteroidota* was significantly decreased following the intervention (Figure S3). Other phyla with statistically significant alteration in abundance by the intervention include *Actinobacteriota*, *Patescibacteria,* and *Desulfobacterota*. Among these, the relative abundances of *Actinobacteriota* and *Patescibacteria* were significantly decreased, and the relative abundance of *Desulfobacterota* was significantly increased (Table 2).

**Table 2:** Alterations in mean relative abundance of bacterial phyla after the intervention

Phylum	Mean relative abundance		*p*-value	
	Baseline	Week 4	*p*	Adjusted *p*
***Firmicutes***	73.6078	60.2637	**0.000295**	0.001473
***Bacteroidota***	16.1290	30.7295	**0.000013**	0.000160
*Proteobacteria*	8.2690	6.7383	0.575194	0.690233
***Actinobacteriota***	0.9746	0.1415	**0.000368**	0.001473
*Fusobacteriota*	0.6284	0.8277	0.796949	0.796949
*Verrucomicrobiota*	0.2746	1.1088	0.300819	0.501151
***Patescibacteria***	0.0609	0.0151	**0.036505**	0.087612
***Desulfobacterota***	0.0524	0.1218	**0.031814**	0.087612
*Campylobacterota*	0.0015	0.0019	0.700477	0.764156
*Fibrobacterota*	0.0013	0.0087	0.379032	0.505376
*Elusimicrobiota*	0.0005	0.0166	0.334101	0.501151
*Cyanobacteria*	0.0000	0.0263	0.129971	0.259941

The bold values emphasize statistical significance.

We next summarized the relative abundance of all taxa with statistically significant differences between groups at the phylum, class, order, family, and genus levels (Table 3). As far as the phyla *Firmicutes* is concerned, the reduction in relative abundance was mainly due to the decrease in class *Clostridia*, orders *Lachnospirales* and *Lactobacillales*, families *Lachnospiraceae* and *Streptococcaceae*; at the genus level, most taxa with higher relative abundance (*Subdoligranulum*, *Ruminococcus*, *Agathobacter*, *[Eubacterium] _hallii_group,* and *Streptococcus*) at baseline were significantly decreased by 4 weeks of intervention. Notably, among the *Firmicutes* phyla, where the relative abundance was significantly decreased by 4 weeks of intervention, several low-abundance genera (*Flavonifractor*, *Oscillibacter*, *UCG_003*, *Anaerotruncus*, *Tuzzerella,* and *Fusicatenibacter*) were significantly increased by the intervention. As for the phyla *Bacteroidota*, the increment in relative abundance was due to the increase in class *Bacteroidia*, order *Bacteroidales*, families *Bacteroidaceae*, *Tannerellaceae*, and *Marinifilaceae*; at the genus level, the relative abundance of *Paraprevotella*, *Bacteroides*, *Parabacteroides,* and *Odoribacter* were all consistantly increased. Likewise, reduction of the relative abundance in phyla *Actinobacteriota* was mainly owing to a decrease in class *Actinobacteria*, orders *Bifidobacteriales*, *Micrococcales* and *Actinomycetales*, families *Bifidobacteriaceae*, *Micrococcaceae,* and *Actinomycetaceae*; at the genus level, relative abundance of *Actinomyces*, *Bifidobacterium,* and *Rothia* were all reduced. In the case of *Patescibacteria* phyla, the reduction in its relative abundance was due to the decrease in the relative abundance of class *Saccharimonadia*, order *Saccharimonadales*, family *Saccharimonadaceae*, and genus *TM7x*. The least abundant phylum with significant differences among the baseline and week-4 groups is *Desulfobacterota*, where the relative abundance is significantly increased due to the enrichment in class *Desulfovibrionia*, order *Desulfovibrionales*, and family *Desulfovibrionaceae*.

**Table 3:** Significantly (*p* < 0.05) altered taxa from phylum to genus levels after 4 weeks of HPLC intervention

Taxon	Annotation	Mean relative abundance		Change	*p*-value	
		Baseline	Week 4		*p*	Adjusted *p*
*Firmicutes*	Phylum	73.6078	60.2637	↓	0.000295	0.001473
*Clostridia*	Class	57.5296	47.5586	↓	0.007759	0.041380
*Subdoligranulum*	Genus	4.7349	2.5578	↓	0.037976	0.474618
*Ruminococcus*	Genus	2.3861	0.5015	↓	0.003707	0.168756
*Flavonifractor*	Genus	0.0357	0.1969	↑	0.007047	0.192020
*Oscillibacter*	Genus	0.0332	0.1147	↑	0.036549	0.474618
*UCG_003*	Genus	0.0185	0.0895	↑	0.004645	0.168756
*[Eubacterium]_nodatum_group*	Genus	0.0083	0.0009	↓	0.006562	0.192020
*Anaerotruncus*	Genus	0.0010	0.0092	↑	0.039813	0.474618
*Lachnospirales*	Order	20.5601	14.5661	↓	0.015695	0.156947
*Lachnospiraceae*	Family	20.5578	14.5636	↓	0.015699	0.349297
*Agathobacter*	Genus	5.8101	0.7451	↓	0.004439	0.168756
*[Eubacterium]_hallii_group*	Genus	0.1005	0.0569	↓	0.037492	0.474618
*Tuzzerella*	Genus	0.0153	0.0488	↑	0.040970	0.474618
*Lactobacillales*	Order	2.5214	0.4689	↓	0.040285	0.201426
*Streptococcaceae*	Family	1.3174	0.3774	↓	0.034315	0.374262
*Streptococcus*	Genus	1.3163	0.3747	↓	0.033824	0.474618
*Fusicatenibacter*	Genus	0.2219	0.4248	↑	0.018549	0.449301
*Bacteroidota*	Phylum	16.1290	30.7295	↑	0.000013	0.000160
*Bacteroidia*	Class	16.1290	30.7295	↑	0.000013	0.000213
*Bacteroidales*	Order	16.0795	30.7247	↑	0.000013	0.000520
*Paraprevotella*	Genus	0.1123	0.3300	↑	0.043543	0.474618
*Bacteroidaceae*	Family	10.1138	15.4874	↑	0.042052	0.374262
*Bacteroides*	Genus	10.1138	15.4874	↑	0.042052	0.474618
*Tannerellaceae*	Family	2.2115	9.0084	↑	0.000363	0.026717
*Parabacteroides*	Genus	2.2115	9.0084	↑	0.000363	0.065442
*Marinifilaceae*	Family	0.1126	0.2400	↑	0.020884	0.371738
*Odoribacter*	Genus	0.0750	0.1695	↑	0.023405	0.474618
*Actinobacteriota*	**Phylum**	0.9746	0.1415	↓	0.000368	0.001473
*Actinobacteria*	Class	0.9103	0.1103	↓	0.000498	0.003984
*Actinomyces*	Genus	0.0137	0.0009	↓	0.000901	0.065442
*Bifidobacteriales*	Order	0.8521	0.1002	↓	0.000609	0.012008
*Bifidobacteriaceae*	Family	0.8521	0.1002	↓	0.000609	0.026717
*Bifidobacterium*	Genus	0.8443	0.0960	↓	0.000665	0.065442
*Micrococcales*	Order	0.0445	0.0092	↓	0.028809	0.201426
*Micrococcaceae*	Family	0.0445	0.0092	↓	0.028809	0.374262
*Rothia*	Genus	0.0445	0.0092	↓	0.028809	0.474618
*Actinomycetales*	Order	0.0137	0.0009	↓	0.000901	0.012008
*Actinomycetaceae*	Family	0.0137	0.0009	↓	0.000901	0.026717
*Patescibacteria*	**Phylum**	0.0609	0.0151	↓	0.036505	0.087612
*Saccharimonadia*	Class	0.0609	0.0151	↓	0.036505	0.116816
*Saccharimonadales*	Order	0.0609	0.0151	↓	0.036505	0.201426
*Saccharimonadaceae*	Family	0.0566	0.0124	↓	0.041233	0.374262
*TM7x*	Genus	0.0471	0.0067	↓	0.037719	0.474618
*Desulfobacterota*	**Phylum**	0.0524	0.1218	↑	0.031814	0.087612
*Desulfovibrionia*	Class	0.0524	0.1218	↑	0.031814	0.116816
*Desulfovibrionales*	Order	0.0524	0.1218	↑	0.031814	0.201426
*w*	Family	0.0524	0.1218	↑	0.031814	0.374262

“↓” denotes reduction in relative abundance and “↑” indicates upregulation in relative abundance.

### 3.5 LEfSe analysis of GMB taxonomic biomarkers

**Figure 4:**
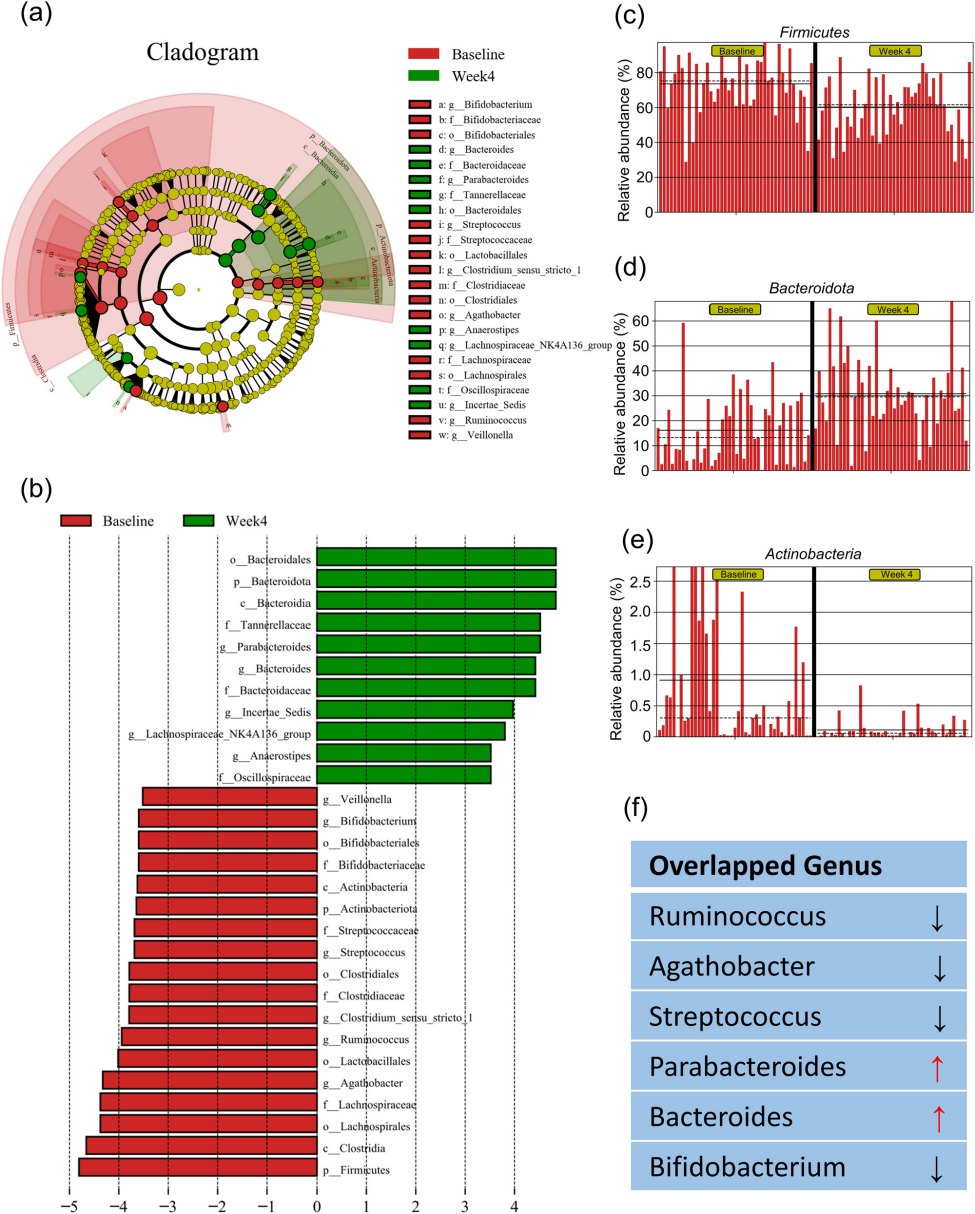
LEfSe analysis of gut microbiota taxonomic biomarkers. (a and b) Cladogram and LDA bar column plotted from LEfSe analysis. The logarithmic LDA score threshold was 3.5 for discriminative features. (c–e) Bar graphs showing changes in relative abundance of three differentially enriched phylum. The solid and dashed lines show the mean and median relative abundance values of each taxon. (f) Overlapped genus between ANOVA and LEfSe analyses and their changes after LCD intervention. “↓” denotes reduction in relative abundance and “↑” indicates upregulation in relative abundance.

Based on the above results, we further performed LEfSe to identify core taxa contributing to the differences between the baseline and week-4 time points. We set the LDA threshold at 3.5. This analysis showed three phyla, three classes, five orders, seven families, and ten genera to be differentially enriched between the baseline and week-4 groups (Figure 4a and b). The relative abundance of the three phyla in every sample is shown in Figure 4c–e, displaying a significant reduction in *Firmicutes* and *Actinobacteriota*, along with a significant increase in the relative abundance of *Bacteroidota* at week 4 compared to the baseline.

To ensure a comprehensive analysis, we considered the intersection of the results obtained from both ANOVA and LEfSe analysis. As ANOVA and LEfSe employ different algorithms to identify differentially enriched taxa between the baseline and week 4, combining their findings provided a more robust and reliable assessment. This analysis identified three phyla (*Bacteroidota*, *Firmicutes,* and *Actinobacteriota*), three classes (*Bacteroidia*, *Clostridia,* and *Actinobacteria*), four orders (*Bacteroidales*, *Lachnospirales*, *Lactobacillales,* and *Bifidobacterials*), five families (*Tannerellaceae*, *Bacteroidaceae*, *Lachnospiraceae*, *Streptococcaceae*, and *Bifidobacteriaceae*) and six genera (*Ruminococcus*, *Agathobacter*, *Streptococcus*, *Parabacteroides*, *Bacteroides,* and *Bifidobacterium*) (Table 4; Figure 4f). Relative abundance of the six genera in every sample is shown in Figure S4, which displayed a robust reduction in levels of *Ruminococcus*, *Agathobacter*, *Streptococcus*, and *Bifidobacterium*, and a steady increase in levels of *Parabacteroides* along with *Bacteroides*. Taken together, the abovementioned taxa could be used as potential biomarkers behind the effectiveness of short-term LCD intervention.

**Table 4:** Summary of overlapped taxa from ANOVA and LEfSe analyses

Phyla	Class	Order	Family	Genus
*Firmicutes↓*	*Clostridia↓*			*Ruminococcus↓*
		*Lachnospirales↓*	*Lachnospiraceae↓*	*Agathobacter↓*
		*Lactobacillales↓*	*Streptococcaceae↓*	*Streptococcus↓*
*Bacteroidota↑*	*Bacteroidia↑*	*Bacteroidales↑*	*Tannerellaceae↑*	*Parabacteroides↑*
			*Bacteroidaceae↑*	*Bacteroides↑*
*Actinobacteriota↓*	*Actinobacteria↓*	*Bifidobacteriales↓*	*Bifidobacteriaceae↓*	*Bifidobacterium↓*

“↓” denotes reduction in relative abundance and “↑” indicates upregulation in relative abundance.

## 4 Discussion

GMB is a highly complex community of microorganisms that reside in the gastrointestinal tract and play a crucial role in digestion [27]. Growing evidence indicates that the composition, functions, balance, and interactions of the GMB with the host have a significant impact on human health [25,39]. Human GMB primarily consists of four major phyla: *Bacteroides*, *Firmicutes*, *Proteobacteria*, and *Actinobacteria*, which collectively account for over 98% of the microbiome [25]. Previous studies have estimated that the human GMB weighs approximately 1–2 kg and is comprised of over 1,000 bacterial species, harboring more than 9 million genes [40,41]. Remarkably, the number of bacterial cells in the gut surpasses that of human cells in the body by a factor of 10 [42]. These findings collectively emphasize the critical role of the GMB in maintaining human health.

In this before–after study, a 4-week intervention with an LCD diet resulted in significant reductions in body weight, BMI, body fat, and visceral fat area, accompanied by a decrease in total cholesterol levels. These findings underscore the effectiveness of the intervention. Moreover, the analysis of 16S rRNA gene sequencing revealed a substantial increase in the diversity of bacterial taxa in each sample following the intervention. Through the integration of ANOVA and LEfSe analyses, we identified several microbiota taxa that responded to the LCD intervention, including three phyla, three classes, four orders, five families, and six genera. Among the three phyla, relative abundances of *Firmicutes* and *Actinobacteriota* were decreased significantly and that of *Bacteroidetes* was increased significantly. At the genus level, we found a significant reduction in relative abundances of *Ruminococcus*, *Agathobacter*, *Streptococcus,* and *Bifidobacterium*, along with a steady increase in relative abundance of *Parabacteroides* and *Bacteroides*. In the following sections, we will discuss the associations of these taxa with obesity and diet.

### 4.1 *Firmicutes* to *Bacteroidetes* ratio, diet, and weight loss

Extensive research activities have been dedicated to identifying the bacterial taxa associated with the onset and control of obesity [43]. It has been demonstrated that the GMB of obese humans or animals exhibits a characteristic higher *Firmicutes* to *Bacteroidetes* (F/B) ratio than normal-weight counterparts, suggesting this ratio as a biomarker of obesity [44,45]. This may be explained by the fact that a 20% increase in the relative abundance of *Firmicutes* and a corresponding decrease in *Bacteroidetes* are associated with an increased energy harvest of around 150 kcal [46]. Additionally, research suggests that *Firmicutes* may promote more efficient energy extraction from food [47]. The byproducts of this process, short-chain fatty acids, can contribute to fat storage and weight gain. When obese people lose weight, the F/B ratio was suggested to decrease [44]. In line with this notion, a decreased abundance of *Firmicutes* over *Bacteroidetes* and *Bifidobacteria* is suggested to be beneficial in preventing and treating obesity [48]. In our study, consistent with previous findings, a high F/B ratio was observed among the study participants at the beginning. Likewise, following a 4-week intervention with an LCD diet, a significant decrease in the F/B ratio was observed (*p* < 0.001) (Figure S3). It is important to note that some studies have failed to establish this relationship [49,50]. Potential factors contributing to these conflicting results include variations in geographical location, weather conditions, regional dietary preferences, duration and composition of interventions, and differences in sample collection and processing methods among other factors. These discrepancies warrant further investigation [51].

### 4.2 Ruminococcus, Agathobacter, Streptococcus, diet, and weight loss

The *Firmicutes* phylum is considered a significant bacterial group associated with obesity, as mentioned earlier. Within the *Firmicutes* phylum, there are over 200 different genera of Gram-positive organisms, primarily responsible for butyrate production in the colon [52]. Studies have shown that germ-free mice, devoid of GMB, have lower body fat compared to mice with a normal GMB, even when subjected to a high-calorie diet [29]. Moreover, the abundance of *Firmicutes* is positively correlated with obesity levels in these mice. Although the exact mechanisms through which *Firmicutes* contribute to obesity are not yet fully understood, it is hypothesized that they play a role in increasing energy extraction from the diet [52]. Consequently, it is crucial to further investigate the specific changes in members of this phylum following a weight-loss diet.

In this study, we identified three genera, including *Ruminococcus*, *Agathobacter*, and *Streptococcus*, all of which belong to the order *Clostridia* (Table 4), to be significantly decreased following short-term LCD intervention. *Ruminococcus* is reportedly associated with increased absorption of sugars via the breakdown of cellulose (with the formation of methane), which may also contribute to weight gain through sugary foods [53]. Supporting the role of *Ruminococcus* in obesity, another study also reported an increased abundance of this bacterial taxa in obese mice fed with a high-fat diet [54]. Like most taxa in the *Firmicutes* phylum, *Agathobacter* is also a butyrate-producing bacterial genus, the increased abundance of which contributes to obesity. A study reported the enrichment of *Agathobacter* in the non-alcoholic fatty liver disease group in comparison with the healthy controls [55], suggesting the involvement of this genus in adiposity. In line with our findings, another study also reported a significant reduction in the relative abundance of *Agathobacter* following caloric restriction of obese women [56]. The *Streptococcus* genus might play important roles in inflammation and comorbid pneumonia among obese individuals. A previous report indicated that adults with grade-3 morbid obesity (BMI ≥ 40 kg/m^2^) had a 15-fold higher relative risk of invasive group A *Streptococcus* infection compared to those of normal weight [57]. Hence, reducing the levels of *Streptococcus* through LCD intervention in our study may be advantageous in reducing inflammation. Further investigation is warranted to explore the roles of the aforementioned three genera in dietary calorie intake.

### 4.3 Parabacteroides, Bacteroides, diet, and weight loss

The *Bacteroidetes* phylum, typically abundant in lean individuals, exhibits decreased relative abundance in the context of obesity. Several studies have reported an increased level of *Bacteroidetes* in obese individuals following weight-loss interventions [44,45]. The *Bacteroidetes* phylum encompasses approximately 20 genera of Gram-negative bacteria, which predominantly produce acetate and propionate in the colon [52]. They are associated with lower energy extraction from the diet compared to *Firmicutes* [46]. The higher abundance of *Bacteroidetes* observed in vegetarians and vegans further supports this notion [58]. While most studies focus on changes in the overall abundance of the *Bacteroidetes* phylum after dietary interventions, specific attention to changes in the levels of genera within this phylum has been limited. Our study revealed a robust increase in the relative abundances of *Parabacteroides* and *Bacteroides*. In line with our findings, *Parabacteroides distasonis* has been demonstrated to alleviate obesity and metabolic dysfunctions through the production of succinate and secondary bile acids [59]. *Parabacteroides goldsteinii* was shown to exert anti-obesity effects in mice [60]. Additionally, an increased abundance of *Parabacteroides merdae* has been found to protect against obesity-associated atherosclerosis by enhancing branched-chain amino acid catabolism [61]. Furthermore, the gut commensal *Bacteroides acidifaciens* reportedly prevents obesity and improves insulin sensitivity in mice [62], while *Bacteroides* have been shown to promote branched-chain amino acid catabolism in brown fat and inhibit obesity in mice [63]. Collectively, these studies support the beneficial role of *Parabacteroides* and *Bacteroides* in improving obesity following dietary interventions in our study.

### 4.4 *Bifidobacterium*, diet, and weight loss

The *Actinobacteria* phylum, although representing only a small percentage, is one of the four major phyla of the GMB. Within this phylum, *Bifidobacterium* has been frequently implicated in obesity. However, the role of *Bifidobacterium* in obesity is somewhat contradictory [64]. On the one hand, a notable increase in the number of *Bifidobacterium* has been observed in obese mice supplemented with inulin-type fructans [65]. On the other hand, there is an inverse correlation between the number of *Bifidobacterium* and the development of fat mass, glucose intolerance, and lipopolysaccharide levels in high-fat diet-induced diabetic mice [66]. Additionally, calorie restriction in mice has been shown to significantly increase the relative abundance of *Bifidobacterium* [67]. Here, we noted a significant decrease in the relative abundance of *Bifidobacterium* following LCD-mediated weight loss. The discrepancies in these findings could potentially be explained by differences in dietary content and intervention duration. Consequently, these findings suggest that individuals with obesity should exercise caution when consuming *Bifidobacterium*-containing prebiotics.

### 4.5 Implications for individualized therapy of obesity

Obesity is a complex condition influenced by multiple factors, each with its unique dimensions. Hence, one-size-fits-all approaches cannot serve as definitive solutions for effectively addressing obesity. A personalized therapy approach, in line with the current trend in the medical field, is being embraced by basic and clinical researchers as well as patients, particularly in the context of obesity [68]. To successfully implement personalized therapy for obesity, it is crucial to acquire comprehensive information regarding each individual patient. Stratifying obese patients based solely on BMI has its limitations [69]. Factors such as ethnicity, age, sex, and muscle mass can influence the interpretation of body fat levels explained by BMI. Therefore, the introduction of novel biomarkers is essential to better characterize obese patients and facilitate personalized therapy. Fortunately, advancements in sequencing and molecular taxonomic methodologies have made it possible to detect the abundance of specific microbial taxa or even map the entire microbiome fingerprints of individuals. This invaluable information can provide crucial guidance and serve as a theoretical basis for personalized targeted therapy in obesity [70]. In the current study, we observed significant changes in several taxa of the GMB following LCD intervention, which could potentially mediate the weight loss achieved. In the future, specific bacterial taxa may be directly targeted as part of personalized therapy to combat obesity.

In this study, we focused on the effects of short-term LCD intervention on obesity and GMB. Long-term LCDs are also a common strategy for weight loss and blood sugar control [71,72]. Intriguingly, a long-term LCD intervention study reported that microbiota shows resilience (a tendency to return to the initial state) to alterations from the diets despite significant weight loss [73]. However, strict adherence to a long-term LCD may lead to adverse health effects. These can include nutrient deficiencies due to limited intake of important nutrients found in carbohydrate-rich foods like whole grains, fruits, and certain vegetables [74]. Digestive issues like constipation or diarrhea may arise from reduced fiber intake. Muscle loss could occur as a result of severely restricting carbohydrates. Additionally, the long-term sustainability of LCDs may be a concern due to strict dietary restrictions and social limitations, which could impact weight loss or overall health goals [75]. Therefore, it is crucial to seek guidance from healthcare professionals or registered dietitians before making LCD-mediated interventions for obese/overweight individuals.

### 4.6 Study limitations

Conducting a single-arm study without a control group has some limitations. First, the absence of a control group makes it difficult to determine if the observed changes in the participants’ microbiome are truly attributed to the LCD intervention or if they are due to other factors. Second, without a control group, it is challenging to establish a baseline for comparison, making it hard to quantify the magnitude of the observed changes. Third, the lack of a control group hinders the ability to account for potential confounding variables that might influence the results, such as participants’ lifestyle, diet, or other concurrent interventions. In order to minimize potential confounding factors and identify the microbial taxa that were consistently associated with the LCD intervention, we took the intersection of LEFse and ANOVA analyses when analyzing and further discussing differentially enriched microbiota.

## 5 Conclusions

In this before–after study, short-term intervention of 4 weeks with LCD resulted in significant reductions in body weight, BMI, body fat, and visceral fat area. Additionally, there was a decrease in total cholesterol levels, indicating the effectiveness of the intervention. The analysis of 16s rRNA gene sequencing data demonstrated a significant increase in the richness of the microbiota following the intervention. By utilizing one-way ANOVA and LEfSe analyses, we identified several microbiota taxa that responded to the LCD intervention. This included three phyla, three classes, four orders, five families, and six genera. Notably, the relative abundance of *Firmicutes* showed a significant decrease, while that of *Bacteroidetes* and *Actinobacteriota* showed a significant increase at the phylum level. At the genus level, *Ruminococcus*, *Agathobacter*, *Streptococcus*, and *Bifidobacterium* exhibited a significant reduction in relative abundance, whereas *Parabacteroides* and *Bacteroides* showed a steady increase. As a whole, our findings demonstrate that LCD can effectively alleviate obesity and induce changes in specific taxa of the GMB. These changes may be involved in LCD-mediated weight loss and provide potential insights for personalized dietary interventions targeting obesity.

While our study demonstrates that LCD can effectively induce weight loss and result in significant changes in the GMB, it is important to mention potential side effects related to LCD, especially in the case of long-term intervention [74]. Thus, we recommend to seek guidance from healthcare professionals or registered dietitians during LCD intervention. Our study also sheds light on the important role that the GMB plays in weight management. However, the specific mechanisms through which the microbiome changes affect body weight and metabolism still require further elucidation. Its impact on host metabolism, energy harvest, appetite regulation, and inflammation remains to be fully clarified. Further, inter-individual variability in diet response strongly suggests the need for future studies considering genetic and environmental factors, and for more targeted, personalized interventions. While we have identified specific microorganisms, such as *Firmicutes*, *Bacteroidetes,* and *Actinobacteriota* as being responsive to LCD, more research should be done to understand their specific roles in weight loss and their potential for utilization in the design of individualized diet plans.

In summary, while our results are encouraging, they represent only a preliminary step toward understanding the complex interactions between diet, GMB, and obesity. Future studies should strive to clarify the underlying mechanisms, examine the long-term effects of these dietary interventions, and explore ways to optimize individual benefits while minimizing potential adverse effects.

## Supplementary Material

Supplementary Figure
