# Supplementary material for: Gut microbiota changes associated with low-carbohydrate diet intervention for obesity
Source: Open Life Sci. 2024 Jan 27;19(1):20220803. doi: 10.1515/biol-2022-0803 (PMC10828666; doi:10.1515/biol-2022-0803)
Supplement: Supplementary Figure [file biol-2022-0803-sm.pdf]

Supplementary Figure Legends and Figures

Figure S1. **Number of OTUs in each sample at baseline and week 4**.

Figure S2. **Changes in *α* and *β* diversity**. (**a** and **b**) Box plots showing alterations in Shannon and Simpson indices. (**c-f**) PERMANOVA and ANOSIM analysis of weighted unifrac and bray Curtis.

Figure S3. **Changes in Firmicutes to Bacteroidetes Ratio at baseline and week 4.**

Figure S4. **Relative abundance of the six genera in every sample overlapped genus between ANOVA and LEfSe analysis**. Bar graphs showing changes in relative abundance of three differentially enriched phylum. The solid and dashed lines show the mean and median relative abundance values of each taxon.

Figure S1

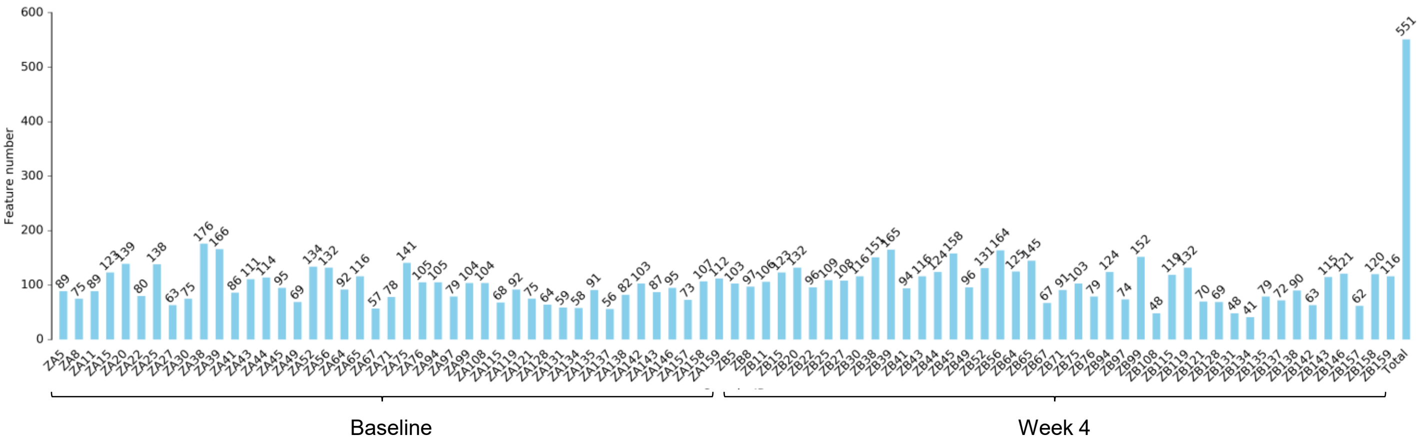

Feature number
600
500
400
300
200
100
0
Total
Baseline
Week 4

Figure S2

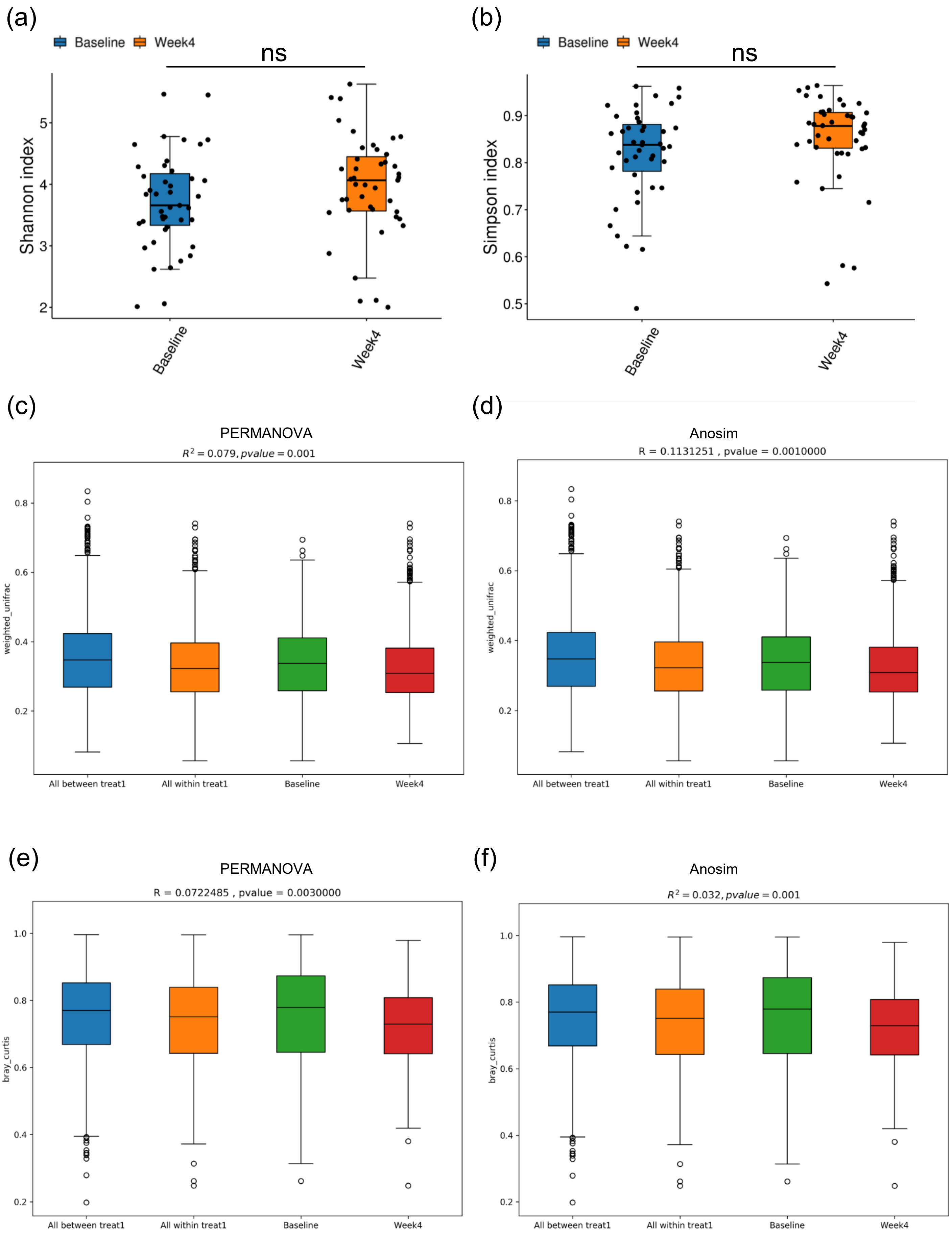

(a)
Baseline
Week4
ns
Shannon index
2
3
4
5
Baseline
Week4
(b)
Baseline
Week4
ns
Simpson index
0.5
0.6
0.7
0.8
0.9
Baseline
Week4
(c)
PERMANOVA
$R^2 = 0.079, pvalue = 0.001$
weighted_unifrac
0.2
0.4
0.6
0.8
All between treat1
All within treat1
Baseline
Week4
(d)
Anosim
R = 0.1131251 , pvalue = 0.0010000
weighted_unifrac
0.2
0.4
0.6
0.8
All between treat1
All within treat1
Baseline
Week4
(e)
PERMANOVA
R = 0.0722485 , pvalue = 0.0030000
bray_curtis
0.2
0.4
0.6
0.8
1.0
All between treat1
All within treat1
Baseline
Week4
(f)
Anosim
$R^2 = 0.032, pvalue = 0.001$
bray_curtis
0.2
0.4
0.6
0.8
1.0
All between treat1
All within treat1
Baseline
Week4

Figure S3

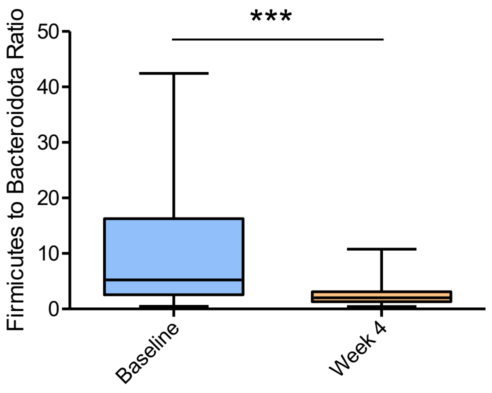

***
Firmicutes to Bacteroidota Ratio
50
40
30
20
10
0
Baseline
Week 4

Figure S4

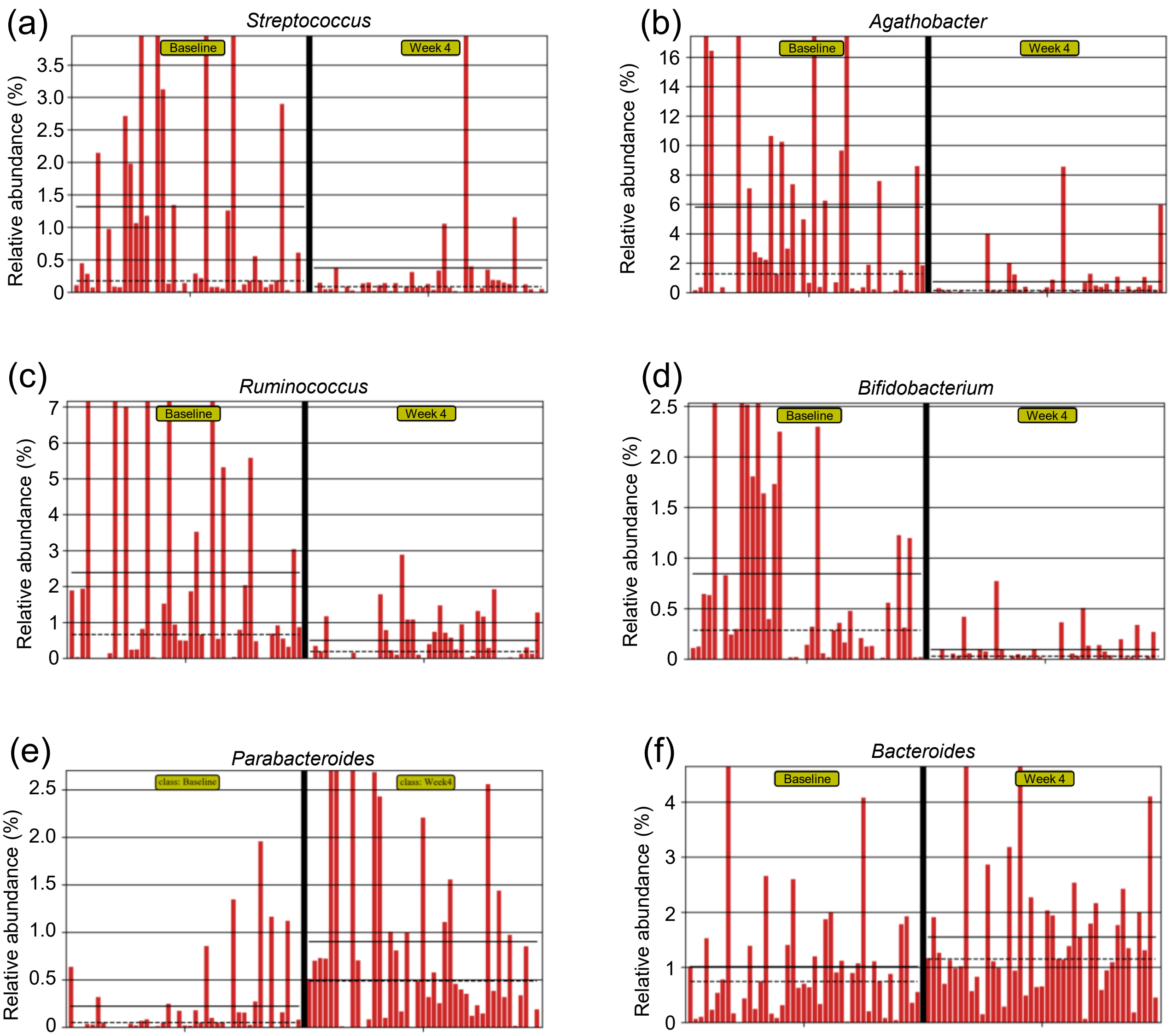

(a)
Streptococcus
Baseline
Week 4
Relative abundance (%)
3.5
3.0
2.5
2.0
1.5
1.0
0.5
0
(b)
Agathobacter
Baseline
Week 4
Relative abundance (%)
16
14
12
10
8
6
4
2
0
(c)
Ruminococcus
Baseline
Week 4
Relative abundance (%)
7
6
5
4
3
2
1
0
(d)
Bifidobacterium
Baseline
Week 4
Relative abundance (%)
2.5
2.0
1.5
1.0
0.5
0
(e)
Parabacteroides
class: Baseline
class: Week4
Relative abundance (%)
2.5
2.0
1.5
1.0
0.5
0
(f)
Bacteroides
Baseline
Week 4
Relative abundance (%)
4
3
2
1
0
